# Analysis of dog movement using a single accelerometer in different body positions—a new approach

**DOI:** 10.3389/fvets.2025.1551341

**Published:** 2025-04-30

**Authors:** Rainer da Silva Reinstein, Franciéli Mallmann Pozzobon, Pâmela Caye, Amanda Oliveira Paraguassú, Brenda Viviane Götz Socolhoski, Otávio Henrique de Melo Schiefler, Ricardo Pozzobon, Daniel Curvello de Mendonça Müller, Maurício Veloso Brun

**Affiliations:** ^1^Postgraduate Program in Veterinary Medicine, Center for Rural Sciences, Federal University of Santa Maria, Santa Maria, Brazil; ^2^Department of Large Animal Clinic, Rural Science Center, Federal University of Santa Maria, Santa Maria, Brazil; ^3^Department of Small Animal Clinic, Rural Science Center, Federal University of Santa Maria, Santa Maria, Brazil; ^4^Research Productivity Scholarship-Level 1C of the Brazilian National Council for Scientific and Technological Development (CNPq) (3304353/2021-3), Santa Maria, Brazil

**Keywords:** gait analysis, biomechanics, accelerometry, dogs, locomotion

## Abstract

**Introduction:**

The analysis of canine locomotion has significantly advanced over the past few decades with the advent of technologies that enable more precise measurements. Traditional methods, such as force platforms and three-dimensional kinematic systems, though accurate, are often costly and require specialized equipment, limiting their broader application. This study aims to evaluate an alternative approach using a single triaxial accelerometer positioned in different anatomical regions (neck, sternum, pelvis, and right knee) to analyze gait patterns in healthy dogs.

**Methods:**

Twenty-four clinically healthy dogs were used, divided into two groups based on body weight: ≤ 15 kg (G−15) and >15 kg (G+15). A wireless triaxial accelerometer sensor was utilized. Acceleration data were collected during walking and trotting in different anatomical positions: neck, sternum, pelvis, and right knee. The data were processed using Fourier analysis to extract harmonic frequencies and analyzed for acceleration peaks and autocorrelation to assess gait symmetry.

**Results:**

The findings showed that larger and heavier dogs (G+15) exhibited lower movement frequencies and more stable patterns, especially during trotting, while smaller and lighter dogs (G−15) demonstrated higher frequencies and greater variability. Significant differences in acceleration peaks were observed between body regions, with the pelvis and knee showing the highest values. However, harmonic frequencies did not vary significantly between the different anatomical regions. The autocorrelation analysis revealed that, in larger dogs, the sternum and pelvis regions presented greater consistency, indicating enhanced stability during locomotion.

**Discussion:**

These findings suggest that using a single accelerometer in different body regions is a practical and effective methodology for gait analysis in dogs, allowing the identification of locomotion differences among dogs of varying sizes and movement phases. This approach offers an accurate alternative for veterinary biomechanical studies, with potential clinical applications in the diagnosis and monitoring of gait abnormalities. The use of a single triaxial accelerometer proved effective for canine gait analysis, revealing differences by body weight. The sternum and pelvis are ideal monitoring regions, suggesting applications in biomechanical and clinical studies.

## 1 Introduction

In recent decades, humans have focused research efforts on understanding animal gait, leading to the publication of numerous studies (1). For many years, direct observation of animals during locomotion, resulting in a subjective gait assessment, was widely used, making this method less reliable (2). Subsequently, imaging equipment was introduced for this purpose, marking the entry of technology into gait analysis and significantly improving its reliability. The first technique was cinematography, which combined filming and photography, allowing researchers to study walking patterns (3). Today, computational assistance has enabled the quantitative definition of gait characteristics (2). Thus, analyses have been used across various species to assess lameness, monitor its progression, and evaluate responses to clinical or surgical treatments (4).

Advances have enabled the division of gait studies into kinetic and kinematic analyses, with kinetics responsible for quantifying forces and kinematics for quantifying the locomotion cycle (5). Currently, kinetic and kinematic data acquisition primarily relies on multiple force platforms and three-dimensional computer-based kinematic analyses (2, 4, 6). This setup can provide precise and comprehensive data; however, it is costly and limited to specialized practices and/or academic research institutions, making gait analysis impractical due to the financial or spatial constraints of these technologies (2, 4, 7). Nonetheless, the expansion of gait analysis, especially in diagnostic precision and recovery reports for patients with locomotor system disorders, has become increasingly notable (8).

In this context, the use of inertial sensors (accelerometers) emerges as a highly advantageous and accessible alternative, allowing field analyses without the need for a laboratory environment (9). Additionally, the simplicity of accelerometers enables real-time data collection, facilitating long-term studies with minimal intervention in the animal's natural behavior and enabling reliable, precise, sensitive, and rapid data collection (4, 8, 10). This more practical and economical approach has the potential to democratize access to gait analysis in dogs, offering a robust solution for researchers and veterinary professionals (8). Furthermore, biomechanical analysis has been extensively explored in the equine literature, particularly due to the importance of locomotion in evaluating athletic performance and musculoskeletal health in these animals (11). However, the applicability of these devices in dogs presents additional challenges, as canine gait biomechanics are influenced by greater phenotypic diversity, variations in anatomical conformation, and differences in locomotor patterns (12) compared to equines (13). Therefore, understanding the specific aspects of using accelerometers in canine gait analysis is essential to validate their applicability and establish reliable protocols for different body sizes and conformations.

In this study, we evaluated the movement of healthy dogs at a walk and a trot using a single accelerometer attached to different body locations, as an alternative to traditional laboratory-based methods for canine gait biomechanical analysis.

## 2 Materials and methods

Data collection was conducted in accordance with the protocol approved by the Animal Use Ethics Committee of the Federal University of Santa Maria (reference number for this project: 7864190624). Twenty-four dogs (*Canis familiaris*—Linnaeus, 1758), mixed-breed dogs (100% mongrel dogs, with no defined pedigree and no direct ancestry from purebred dogs. However, there was variation among individuals, including both dolichocephalic and mesocephalic dogs, with no exclusive predominance of any cranial type in either group. Nevertheless, none of the dogs were brachycephalic), from the clinical-surgical service of the University Veterinary Hospital of the Federal University of Santa Maria were studied, with body weights equal to or <40 kilograms, body condition scores ranging from 3 to 7, and were free of orthopedic or neurological conditions. Half of these dogs were allocated to a group consisting of animals weighing 15 kg or less (G−15), with a mean body weight and standard deviation, withers height, and body condition score of 6.1 kg ± 3.26, 24.25 cm ± 6.27, and 5.25 ± 0.49, respectively. The other half were allocated to a group consisting of animals with more than 15 kg of body weight (G+15), with a mean body weight of 28.75 kg ± 4.15, withers height of 60 cm ± 3.75, and body condition score of 5 ± 0.44. Dogs with musculoskeletal or neurological disorders, degenerative joint disease, conditions associated with fractures, luxations, ligament ruptures, or any condition resulting in altered limb support and gait did not participate in the study, nor did dogs under 1 year of age or those with prior clinical and/or surgical treatment for orthopedic/neurological conditions.

### 2.1 Accelerometer, data collection, and processing

**Figure 1:**
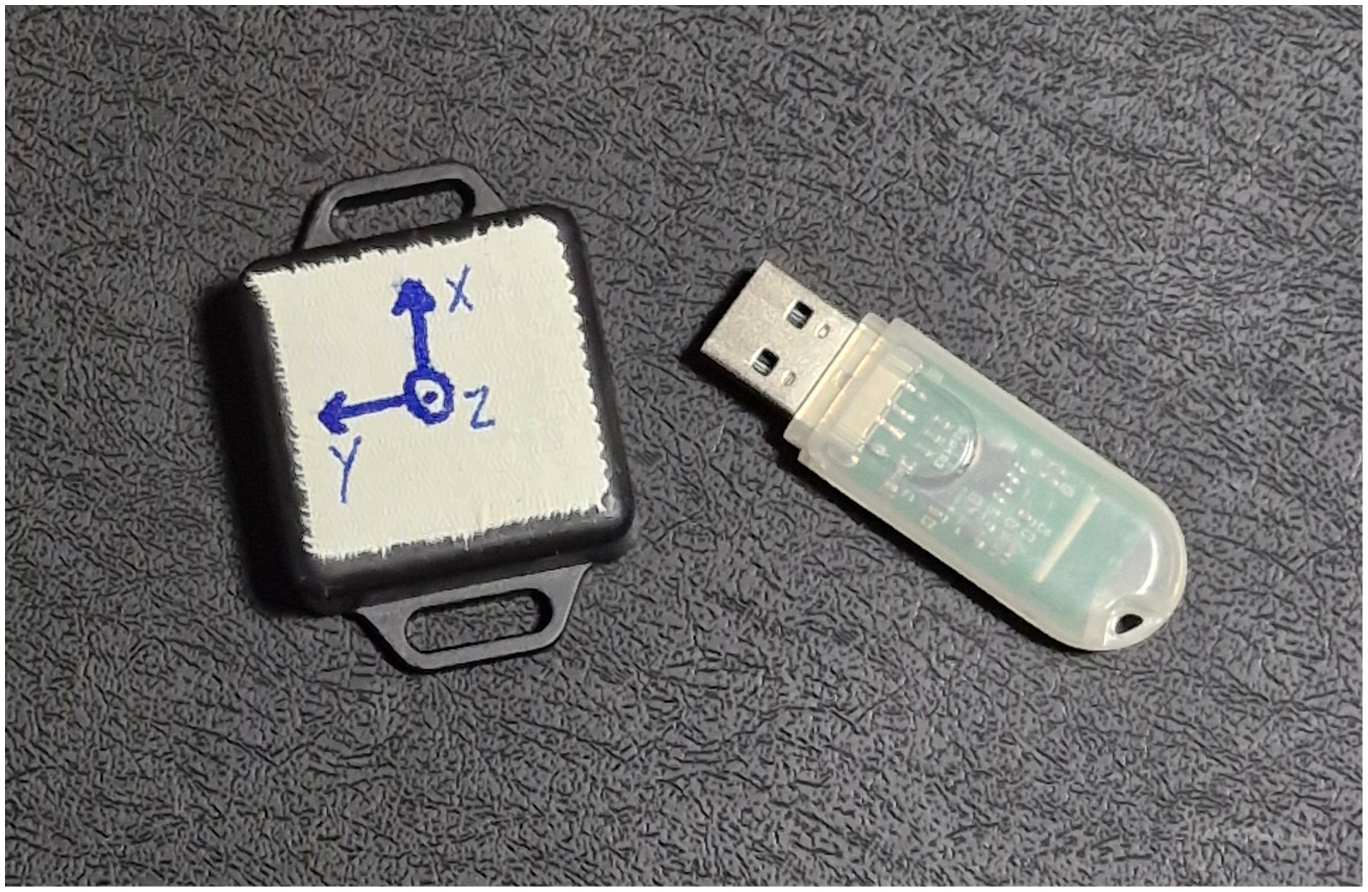
Wireless accelerometer device model BWT901CL (WIT Motion^®^, Shenzhen, China) used in the study for acceleration data collection in dogs, along with its Bluetooth USB receiver model Bluetooth 2.0 USB-HID (WIT Motion^®^, Shenzhen, China). Source: Author.

For acceleration data collection, we used the triaxial inertial sensor (Figure 1) model BWT901CL (WIT Motion^®^, Shenzhen, China). This sensor integrates a triaxial accelerometer, a gyroscope, and a magnetometer, enabling simultaneous measurement of linear acceleration, angular velocity, and spatial orientation. Its physical dimensions are 35 mm (length) × 24 mm (width) × 9 mm (height), with an approximate weight of 10 g, making it light enough not to interfere with the natural movement of the dogs. We positioned the sensor in different regions of the dogs' bodies (neck, sternum, pelvis, and right knee) using adjustable straps (Velcro^®^ Brands, São Paulo, Brazil) or self-adhesive microporous tape, 100 mm × 4.5 m (3M^®^, Sumaré, Brazil), cut as needed to ensure secure attachment without interfering with the animals' mobility. These anatomical positions provided good sensor stability, and the measurement axes varied according to the applied position. The sensor has a 16-bit resolution, ensuring high measurement accuracy, and the sampling frequency is configurable up to 200 Hz, set to 50 Hz in the present experiment to capture movements with sufficient detail for gait analysis.

The BWT901CL sensor was coupled with a compatible Bluetooth receiver (Figure 1), model Bluetooth 2.0 USB-HID (WIT Motion^®^, Shenzhen, China), provided by the same company, for real-time transmission of inertial data to a central computer. The Bluetooth interface enabled a stable wireless connection with a range of up to 10 m, allowing the dogs to move freely during the experiments. The receiver was connected to a laptop via a USB port.

Data were collected in real-time using the MinilMU software, version 6.2.68 (WIT Motion^®^, Shenzhen, China), which allows for sensor reading visualization and data storage in CSV files (comma-separated values). We configured the software to record only the linear acceleration variables in the three axes (X, Y, and Z) in g (gravity) over time, where g = 9.807 m/s^2^. Angular velocity and spatial orientation data were disabled to improve data traffic between the sensor and receiver. Sensor readings were collected continuously during the dogs' walking and trotting phases and were subsequently processed for cyclic pattern analysis.

Prior to analysis, we pre-processed the data to ensure the consistency of the measurements. Thus, we performed noise filtering using a filter [SciPy.Signal library version 1.14.1 in Python programming environment version 3.13.0 (Python^TM^, Python Software Foundation, Wilmington, USA)] with a cutoff frequency of 20 Hz to reduce high-frequency noise caused by unexpected vibrations. The data were then organized into acceleration time series for each body region and were equally processed in the Python programming environment using the NumPy library version 2.1.0 and Pandas version 2.2.3. The Python commands used in this analysis were generated with the assistance of artificial intelligence (ChatGPT-4.0, OpenAI, California, USA).

### 2.2 Sensor positioning

**Figure 2:**
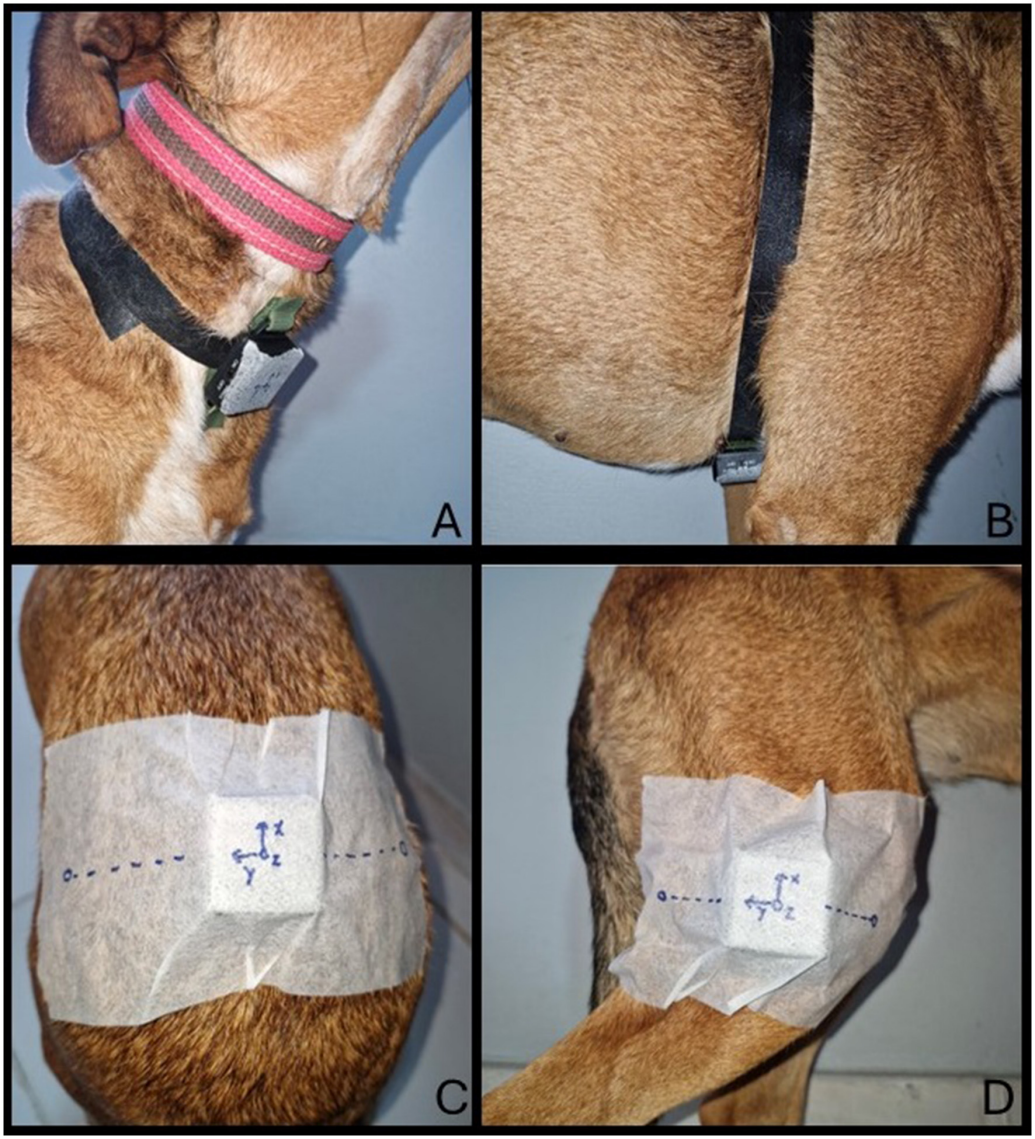
Illustration of accelerometer positions and captured acceleration axes (ax, ay, az) on the dog's body during the study. Sensors were attached to four anatomical regions: neck **(A)**, sternum **(B)**, pelvis**(C)**, and knee **(D)**. Source: Author.

The sensor was always attached by the same investigator, alternately in the following locations (Figure 2).

#### 2.2.1 Neck

Positioned ventrally to capture acceleration associated with head movement. For attachment, the sensor was secured with an adjustable Velcro^®^ strap firmly positioned around the neck, just below the collar, at a distance that prevented any contact between the sensor and the collar. The ax(g) axis recorded acceleration in the caudal-cranial direction, the ay(g) axis recorded acceleration in the left-right direction, and the az(g) axis recorded acceleration in the dorsal-ventral direction.

#### 2.2.2 Sternum

Positioned ventrally at the center of the thorax to capture acceleration resulting from body movement. For attachment, the sensor was secured with an adjustable Velcro^®^ strap firmly positioned around the thorax, just behind the forelimbs. The ax(g) axis recorded acceleration in the caudal-cranial direction, the ay(g) axis recorded acceleration in the left-right direction, and the az(g) axis recorded acceleration in the dorsal-ventral direction.

#### 2.2.3 Pelvis

Positioned on the upper part of the pelvis, between the iliac tuberosities, capturing lumbar region movement during gait. For attachment, the sensor was secured to the pelvis using the iliac tuberosities (midpoint) as an anatomical reference. A self-adhesive microporous tape was used to ensure proper fixation of the sensor in the region. The ax(g) axis recorded acceleration in the caudal-cranial direction, the ay(g) axis recorded acceleration in the right-left direction, and the az(g) axis recorded acceleration in the ventral-dorsal direction.

#### 2.2.4 Right knee

Positioned laterally to the right knee, measuring acceleration associated with the movement of the right pelvic limb. For attachment, the sensor was secured to the lateral side of the right knee, using the tibial tuberosity (midpoint) as an anatomical reference. A self-adhesive microporous tape was used to ensure proper fixation of the sensor in the region. The ax(g) axis recorded acceleration in the ventral-dorsal direction, the ay(g) axis recorded acceleration in the cranial-caudal direction, and the az(g) axis recorded acceleration in the left-right direction.

Data collection was performed while the dogs moved at a walk and a trot on a flat masonry surface, in a straight-line trajectory, covering a minimum distance of 10 meters and recording data for at least 5 s. The same sensor was used for all analyses, and the dogs were always handled by the same conductor using a collar and leash. Data collection was always performed in duplicate, and the average value obtained was considered. The handler controlled the displacement speed to ensure that the evaluated animal maintained a walk or a trot according to the visual characteristics of the gait. However, subsequently, based on the data obtained from the sensor, it was possible to determine that in the G−15 group, the average displacement speed of the dogs while walking, followed by its standard deviation, was 0.9 m/s ± 0.05, and while trotting, it was 1.3 m/s ± 0.03. In the G+15 group, the average displacement speed while walking was 1.2 m/s ± 0.18, and while trotting, it was 1.7 m/s ± 0.11.

## 3 Statistical analysis

**Figure 3:**
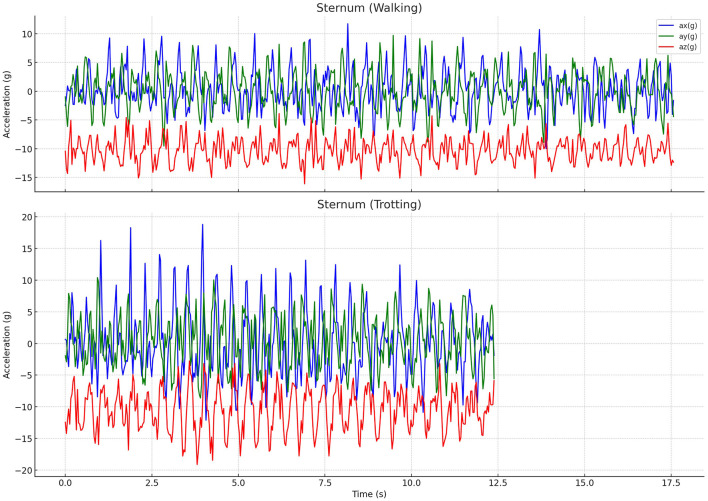
Graphs of acceleration components on three axes (ax, ay, az) during walking and trotting movements in a G+15 dog, measured in the sternum region. The vertical axis represents acceleration in g, while the horizontal axis represents time in seconds. Differences in acceleration behavior between walking (upper part) and trotting (lower part) movements are clearly observable in all three components. Source: Author.

After data acquisition, acceleration vs. time graphs for the X, Y, and Z axes were created for all dogs using Python's Matplotlib library, version 3.9.2. Figure 3 shows these variations at the sternum, during walking and trotting, in one of the G+15 group dogs. Through a visual analysis of the graphs, it was observed that, depending on the anatomical position where the sensor was attached, some acceleration curves were less repetitive or exhibited lower cyclicity. Therefore, we applied Fourier analysis to the accelerometric signals of the Z-axis when the sensor was positioned on the neck, sternum, and pelvis. For the X-axis, readings were obtained when the sensor was positioned on the right knee. Thus, using Python's SciPy library, version 1.14.1, the analysis was performed, and the main movement harmonics were extracted from the Fast Fourier Transform (FFT) spectrum, allowing the squared magnitude of acceleration to be obtained in relation to frequency. Acceleration and deceleration peak analyses were investigated, and averages were calculated. We calculated gait symmetry (at both walk and trot) using an autocorrelation function, allowing us to identify a regular periodic pattern in the gait. All data were compiled into tables, with comparisons made between groups (G−15 and G+15) and by body region where the sensor was applied.

Data normality was assessed using the Shapiro-Wilk test, applied to three main variables: harmonic frequency (derived from Fourier analysis), peak acceleration, and autocorrelation. This test was chosen for its sensitivity in detecting deviations from normality in small samples. Given the absence of normality, non-parametric approaches were adopted: the Mann-Whitney test was used to compare the weight groups (G−15 and G+15), while the Friedman test was employed to evaluate the effect of sensor location within each individual, considering *p* < 0.05 as statistically significant.

## 4 Results

The results obtained from the basic descriptive statistical analysis and the statistical test comparing G−15 vs. G+15 are presented in Tables 1, 2, respectively, while the results of the within-subject analysis, assessing the effect of sensor location within each individual, are described in Table 3. Significant variations can be observed in the variables of harmonic frequency, peak acceleration, and autocorrelation across different body regions and types of movement. The harmonic frequency analysis revealed statistically significant differences between the weight groups for various anatomical regions and types of movement, suggesting that body mass influences the frequency of movement oscillations during canine gait. The within-subject statistical analysis did not reveal statistically significant differences between anatomical regions for harmonic frequency (*p* = 0.993) and autocorrelation (*p* = 0.850), suggesting that these variables are relatively consistent regardless of sensor placement. However, peak acceleration showed a significant difference between anatomical regions (*p* = 0.001), indicating that the accelerometer's location may influence the magnitude of the measured accelerations.

**Table 1:** Mean acceleration values and standard deviation for different body regions of dogs evaluated during walking and trotting movements.

**Region**	**Movement type**	**First harmonic frequency (Hz)**		**First harmonic amplitude (m/s** ^**2**^ **)** ^**2**^		**Peak acceleration (g)**		**First correlation peak**	
		**Mean**	**Standard deviation**	**Mean**	**Standard deviation**	**Mean**	**Standard deviation**	**Mean**	**Standard deviation**
**G**+**15**									
Sternum	Walking	7.87	2.74	7.8	4.37	20.92	17.37	0.41	0.37
Sternum	Trotting	7.28	2.56	11.58	6.91	19.07	14.08	0.28	0.28
Right Knee	Walking	8.55	1.71	7.52	4.18	22.92	23.97	0.43	0.32
Right Knee	Trotting	5.88	2.88	6.17	4.83	20.68	18.57	0.45	0.31
Pelvis	Walking	7.61	2.17	8.27	5.87	21.26	16.29	0.23	0.26
Pelvis	Trotting	7.3	2.21	6.83	4.61	11.42	16.28	0.35	0.3
Neck	Walking	7.74	1.64	7.75	4.29	17.66	15.21	0.35	0.26
Neck	Trotting	7.62	2.65	7.19	4.62	26.9	23.86	0.45	0.35
**G–15**									
Sternum	Walking	10.98	1.35	12.38	9.75	19.91	27.12	0.41	0.39
Sternum	Trotting	10.65	2.37	8.73	4.35	17.11	12.54	0.42	0.25
Right Knee	Walking	10.55	2.57	10.01	10.2	16.13	23.3	0.35	0.3
Right Knee	Trotting	11.16	1.95	9.44	5.68	20.92	17.26	0.57	0.95
Pelvis	Walking	10.99	2.75	6.79	3.76	18.9	25.17	0.42	0.36
Pelvis	Trotting	10.0	2.32	7.54	8.58	30.03	23.15	0.48	0.31
Neck	Walking	10.58	3.81	8.6	6.42	16.71	20.34	0.52	0.26
Neck	Trotting	11.29	2.6	9.25	5.75	22.09	27.67	0.76	1.0

**Table 2:** Results of the Mann-Whitney statistical test between groups for the variables of harmonic frequency, acceleration peak, and autocorrelation, across different anatomical regions (neck, sternum, pelvis, and knee) and types of movement (walking and trotting).

		**Harmonic frequency G**–**15 vs. G**+**15**		**Peak acceleration G**–**15 vs. G**+**15**		**Autocorrelation G**–**15 vs. G**+**16**	
**Region**	**Movement type**	***p*** **-value**	***U*** **-value**	***p*** **-value**	***U*** **-value**	***p*** **-value**	***U*** **-value**
Neck	Walking	^*^0.001	128.0	0.094	42.5	0.488	84.5
Neck	Trotting	^*^0.015	114.5	0.773	66.5	0.133	98.5
Sternum	Walking	^*^0.002	126.5	^*^0.049	37.5	0.214	94.0
Sternum	Trotting	^*^0.003	134.5	0.525	60.5	^*^0.022	32.0
Pelvis	Walking	0.106	100.5	0.862	68.5	^*^0.019	113.0
Pelvis	Trotting	^*^0.001	140.5	0.326	54.5	0.707	65.0
Right Knee	Walking	^*^0.021	112.5	0.840	76.0	0.506	84.0
Right Knee	Trotting	^*^0.003	144.0	0.583	82.0	0.507	84.0

Statistical tests were performed to verify significant differences between groups. *p*-values <0.05 indicate statistical significance. ^*^Significant *p*-value.

**Table 3:** Results of the Friedman test (intra-subject) assessing the effect of accelerometer anatomical location on the variables harmonic frequency, peak acceleration, and autocorrelation.

**Variable**	**p-value**	**df**
Harmonic frequency	0.993	3
Peak acceleration	^*^0.001	3
Autocorrelation	0.850	3

*p* < 0.05 indicate statistical significance. df, degrees of freedom. ^*^Significant p-value.

### 4.1 Neck

Significant differences were found in both walking (*p* = 0.001) and trotting (*p* = 0.015) between the weight groups. The frequency was higher in G+15 (1.75 ± 0.31 Hz in walking and 1.85 ± 0.29 Hz in trotting) compared to G−15 (1.62 ± 0.27 Hz in walking and 1.75 ± 0.26 Hz in trotting).

### 4.2 Sternum

The data for the sternum show that harmonic frequency in walking (*p* = 0.002) and trotting (*p* = 0.003) was significantly different in G+15 (1.88 ± 0.32 Hz in walking and 1.95 ± 0.29 Hz in trotting) compared to G−15 (1.69 ± 0.3 Hz in walking and 1.78 ± 0.27 Hz in trotting).

### 4.3 Pelvis

In trotting (*p* = 0.001), a significant difference was observed between the weight groups (G+15 = 1.9 ± 0.28 Hz and G−15 = 1.72 ± 0.26 Hz), whereas in walking (*p* = 0.106), the difference was not statistically significant.

### 4.4 Right knee

Significant differences were observed in both walking (*p* = 0.021) and trotting (*p* = 0.003), with heavier dogs exhibiting higher harmonic frequencies (1.8 ± 0.31 Hz in walking and 1.92 ± 0.27 Hz in trotting).

The analysis of acceleration peak, which measures the maximum magnitude of acceleration during movement, revealed statistically significant differences in some body regions, although less consistently than harmonic frequency.

### 4.5 Neck

No significant differences were observed between the weight groups in walking (*p* = 0.094) and trotting (*p* = 0.773).

### 4.6 Sternum

A significant difference was found in walking (*p* = 0.049), indicating that lighter dogs tend to exhibit higher acceleration peaks in this region [20.22 ± 3.19 (m/s^2^)^2^] compared to heavier dogs [18.96 ± 3.28 (m/s^2^)^2^].

### 4.7 Pelvis

No significant differences were observed between the weight groups in walking (*p* = 0.862) and trotting (*p* = 0.326).

### 4.8 Right knee

Similarly, no significant differences were observed in walking (*p* = 0.840) and trotting (*p* = 0.583).

Autocorrelation, which assesses the degree of repetition of acceleration patterns during movement, showed mixed results across different body regions.

### 4.9 Neck

No significant differences were observed in walking (*p* = 0.488) and trotting (*p* = 0.133).

### 4.10 Sternum

In walking (*p* = 0.214) and trotting (*p* = 0.020), a significant difference was found only in trotting.

### 4.11 Pelvis

Significant differences were found in both walking (*p* = 0.022) and trotting (*p* = 0.019).

### 4.12 Right knee

No significant differences were observed in walking (*p* = 0.506) and trotting (*p* = 0.507).

## 5 Discussion

The use of inertial sensors, such as accelerometers, is well documented in kinematic studies, particularly in horses (14, 15). Similar devices have been used to assess locomotion and movement asymmetry in horses, demonstrating that these sensors are effective in measuring gait symmetry and detecting lameness, even in subclinical stages (14, 16, 17, 26). In another study (18), the ability of these sensors to detect subtle changes and provide objective data in studies of horse-rider coordination was also observed.

Although much of the literature has focused on horses and specific pathological conditions (14–18), the present study advances the field by using accelerometers for continuous real-time data collection during the gait of healthy dogs. This method allowed for a detailed analysis of gait patterns in different phases (walking and trotting), with distinctions between body weight groups and the assessment of different body regions. Few studies in the veterinary literature have explored this approach, offering a new perspective on canine biomechanical analysis, particularly in clinical applications for monitoring orthopedic and neuromuscular health.

In addition to horses, the use of accelerometers has also been validated in other species, including dogs. Researchers have validated the use of accelerometers in gait analysis for dogs with muscular dystrophy (19), while others have confirmed the validity of these devices for monitoring the habitual physical activity of dogs by comparing the data with video recordings (20). More recently, new research has correlated activity patterns captured by accelerometers with sleep quality in dogs (21). For this purpose, sensors were typically used for long-term monitoring. However, the use of these devices for continuous real-time gait data collection, as performed in the present study, is innovative, as it allows for instant data acquisition using only a single sensor that can be easily attached to the patient's body. This study also determines the optimal location for sensor placement. This approach enables a more detailed and continuous analysis of canine movement in different phases, complementing data from previous studies.

Compared to optical motion capture systems, which are restricted to controlled environments, accelerometers offer a significant advantage: the ability to collect data in outdoor and free-moving environments, as well as reduced costs (22). On the other hand, force platforms, widely used to measure ground reaction forces during gait, are limited to capturing a single step at a time, making it challenging to analyze multiple consecutive steps (5). In contrast, accelerometers enable continuous data capture, providing a more comprehensive view of canine gait under controlled conditions (terrain, presence of a handler, and straight-line movement), while still approximating natural conditions (outside the laboratory environment). Other studies have also found that accelerometers are effective for monitoring activity and movement patterns, highlighting their versatility in different contexts (20, 21).

Fourier analysis, widely used to decompose time series into frequency components, was essential in identifying the main frequencies associated with gait phases (walking and trotting). Previous studies have also used this technique in horses, demonstrating its effectiveness in detecting subtle differences in locomotion patterns (14, 15). Additionally, peak analysis was used to identify moments of highest acceleration during gait, highlighting impact variations between heavier and lighter dogs. This corroborates previous findings that used a similar approach (14, 19). Autocorrelation, in turn, was applied to assess the consistency of gait patterns, showing the regularity of cycles, especially among dogs of different weights. Studies on horses have also used autocorrelation to detect lameness patterns (14, 17).

**Figure 4:**
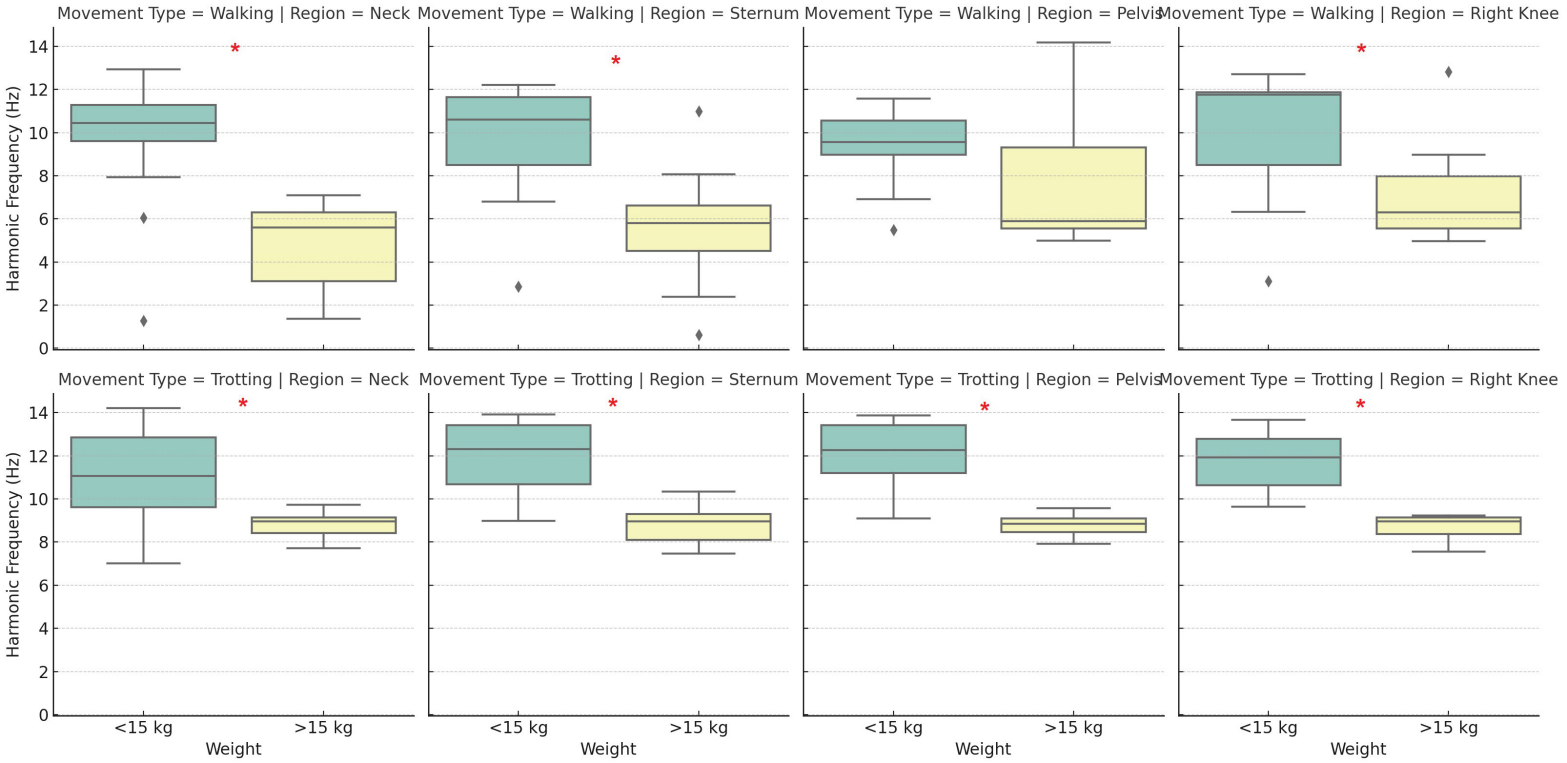
Distribution of harmonic frequencies (in Hz) across different body positions (neck, sternum, pelvis, and knee) between groups, during walking and trotting movements. The boxplot displays the median, quartiles, and data range for each position and movement combination, with possible outliers indicated by circles. Asterisks (*) indicate statistically significant differences (*p* < 0.05) between groups. Source: Author.

Observing the results of the harmonic frequency analysis, it was evident that the influence of body weight varies across different anatomical regions (Figure 4). In the neck, regardless of the type of movement, dogs with higher body mass exhibited significantly different harmonic frequencies compared to lighter dogs, with consistently higher values in heavier dogs. This finding suggests that movement in the neck region is particularly sensitive to variations in body mass, possibly due to the need for head stabilization during locomotion. In the pelvic region, the difference between groups was not statistically significant at the walk but was observed at the trot. This suggests that harmonic frequency in the pelvis is more influenced by body weight during higher-intensity movements, where there is greater mechanical demand and a higher need for postural control. Similarly, knee analysis demonstrated that this region is highly sensitive to variations in body weight, especially during the trot. These findings reinforce the idea that harmonic frequency, which represents the rate of repetitive oscillation during movement, is strongly modulated by body weight, particularly in regions associated with broad and complex movements. The higher harmonic frequency observed in heavier dogs may be related to adaptive locomotion patterns to manage increased mechanical load, suggesting a significant impact of body weight on gait dynamics.

**Figure 5:**
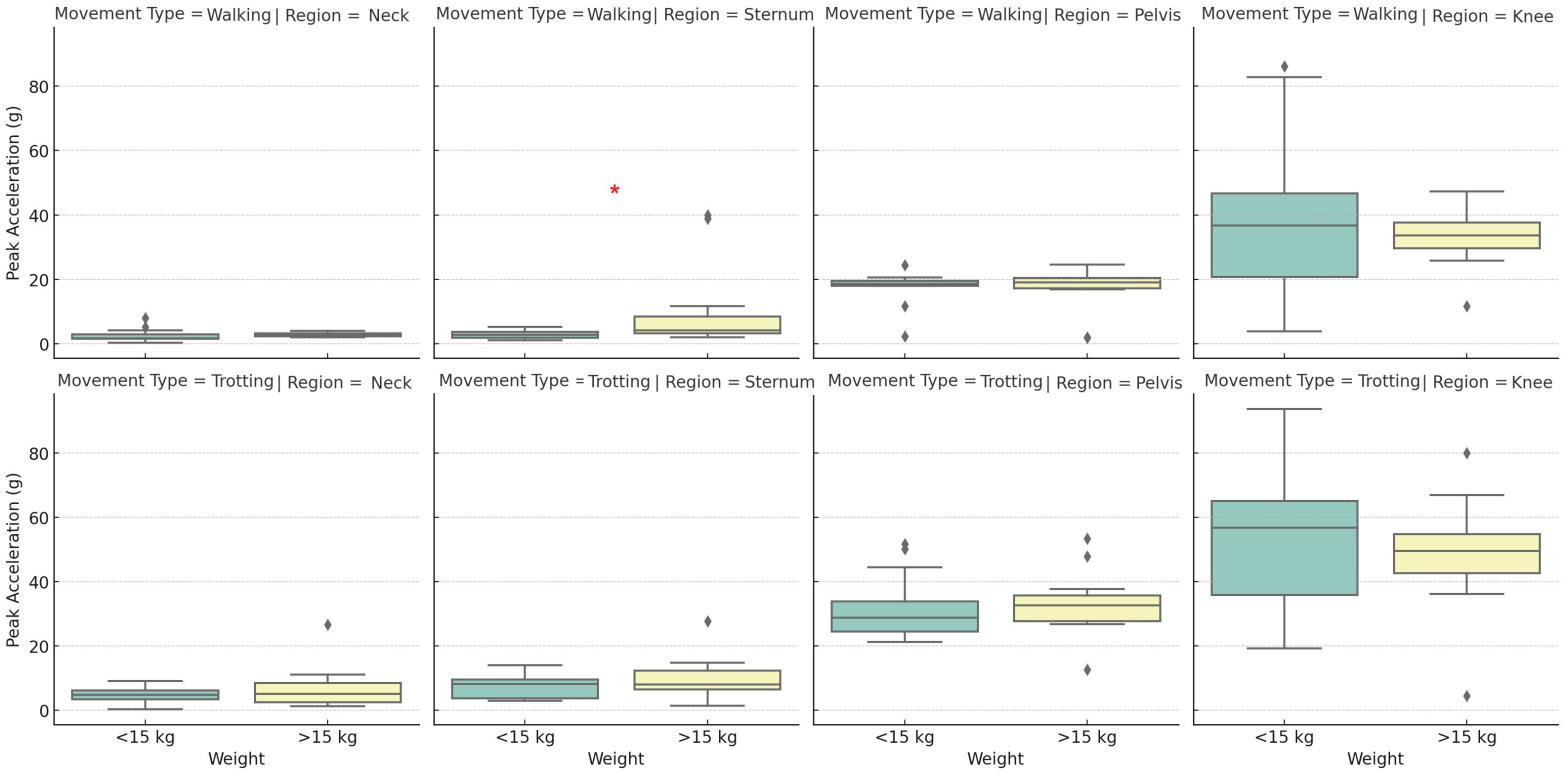
Distribution of acceleration peaks (in g) across different body positions (neck, sternum, pelvis, and knee) between groups, during walking and trotting movements. The boxplot displays the median, quartiles, and data range for each position and movement combination, with possible outliers indicated by circles. Asterisks (*) indicate statistically significant differences (*p* < 0.05) between groups. Source: Author.

The results of the peak acceleration analysis (Figure 5) indicate that this metric is less sensitive to variations in body weight compared to harmonic frequency. In the neck region, no significant differences were observed between groups, suggesting that peak acceleration in this area is not directly influenced by the dogs' body mass. This finding suggests that other factors, such as postural control and cervical motion amplitude, may play a more relevant role in modulating this variable. Similarly, in the pelvic region and the right knee, no significant differences were found between groups, indicating that peak acceleration in these areas is not directly affected by body weight, regardless of the type of movement. These findings suggest that, unlike harmonic frequency, peak acceleration does not exhibit a clear relationship with body weight, making it a metric less influenced by this variable. Few body regions showed significant variations between groups, suggesting that peak acceleration may be more dependent on movement mechanics and the individual locomotor strategy of each dog rather than body weight itself.

The results of the autocorrelation analysis indicate that the degree of repetition in movement patterns varies according to body region and the dogs' weight. In the neck, no significant differences were observed between groups, suggesting that this region maintains a similar movement pattern regardless of body mass. However, in the sternum, a significant difference was identified only during the trot, with heavier dogs showing greater repetition of movement patterns in this region. This suggests that, in dogs with higher body mass, the sternum may play a more stable role in locomotion during high-intensity movements.

**Figure 6:**
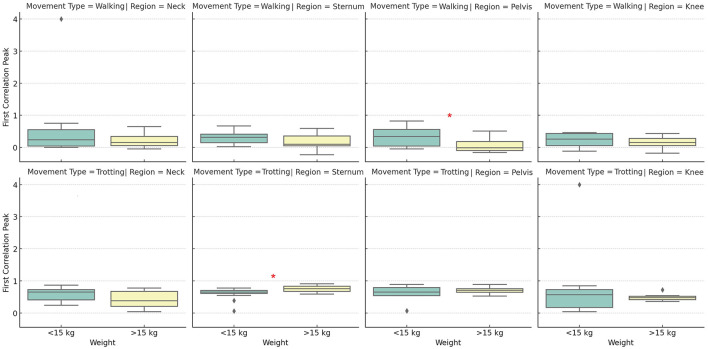
Distribution of autocorrelation coefficients across different body positions (neck, sternum, pelvis, and knee) between groups, during walking and trotting movements. The boxplot displays the median, quartiles, and data range for each position and movement combination, with possible outliers indicated by circles. Asterisks (*) indicate statistically significant differences (*p* < 0.05) between groups. Source: Author.

In the pelvic region, the repetitive movement pattern varied significantly with body weight, indicating that lighter dogs exhibited less repetitive movement in this area, especially during the trot. This finding suggests that the pelvis may be subject to greater postural adjustments in smaller dogs, possibly due to differences in movement biomechanics and impact force distribution. In contrast, in the knee region, no significant differences were observed in either the walk or the trot, suggesting that the repetition pattern of knee movement remains relatively stable regardless of body mass. Overall, the autocorrelation results indicate that the degree of movement pattern repetition can be influenced by body weight, with the pelvis being one of the most sensitive regions to this variable (Figure 6). Additionally, these findings reinforce the idea that harmonic frequency is the metric most consistently affected by body weight across different regions and movement types. In contrast, acceleration and autocorrelation appear to be more locally influenced, depending on the specific body region. These results suggest that body mass plays an important role in canine locomotion dynamics, particularly in regions involved in complex and repetitive movements.

The comparison between different body regions (neck, sternum, pelvis, and knee) revealed that the sternum and pelvis play a central role in impact absorption and stability during locomotion. Therefore, these regions should be considered in gait analysis studies using accelerometry, specifically with the sternum for evaluating forelimb movement and the pelvis for assessing hindlimb motion. Acceleration in the knee region was more variable, reflecting fine adjustments in movement control. The neck showed the least variation in acceleration across all groups, indicating its stabilizing function during movement, regardless of the animal's weight. When comparing the two groups, heavier dogs exhibited a more intense acceleration pattern at both walk and trot (Figure 5), with lower predominant frequencies (Figure 4) and a more erratic pattern at the walk (values closer to zero indicate weaker correlation; Figure 6). Meanwhile, lighter dogs displayed faster accelerations and greater variability in their movement patterns, which may be related to lower efficiency in postural control during the trot. The results suggest that subtle gait alterations observed in a limping dog can be identified through acceleration analysis, as changes in frequency, acceleration peaks, and gait patterns would likely diverge from the values presented in this study. Additionally, during the trot, movement shows greater stability, with the sternum and pelvis playing a crucial role in maintaining this stability. Thus, these areas emerge as the preferred locations for inertial sensor placement, particularly in studies aiming to assess movement patterns in dogs.

We observed that the distribution of force across the acceleration axes varied according to the sensor location and the dogs' body weight. Although the animals evaluated were healthy, body weight variation resulted in biomechanical adaptations that influenced both the force and regularity of gait. Previous studies have reported similar force redistributions in horses with subclinical lameness (16, 17). Our study focused on the analysis of vertical acceleration, which allowed us to assess force variations along this axis during gait. Heavier dogs exhibited higher vertical acceleration values, consistent with increased vertical force due to greater body mass. This metric is crucial for understanding vertical impact and its implications for gait biomechanics, serving as an important complement to studies analyzing multiple movement axes (19).

Despite the promising results, the present study has some limitations. The group division was based exclusively on body weight, following previous biomechanical gait evaluation studies where the importance of weight was clearly demonstrated (23, 24). All dogs were mixed-breed, with various breed influences, which may contribute to biomechanical variation due to phenotypic diversity. The sample size was relatively small, which may have impacted the representativeness of the data, particularly regarding variability among animals and body conformations. Future studies with a larger and more diverse sample would be beneficial to validate our findings. Additionally, sensor placement can influence acceleration results, as suggested by other researchers (20, 21). Although we standardized sensor placement, minor variations in positioning may have affected the data. Specific software was also not used to detect gait alterations resulting from sensor usage. Future studies could investigate the effects of small variations in sensor positioning in more detail.

Another aspect to consider is that, although we collected data in real time, the testing environment was controlled and limited to straight-line steps on homogeneous surfaces. Future studies could include gait analysis on different surfaces and more dynamic contexts, such as uneven terrain or turns, to assess whether the observed patterns remain consistent. Additionally, a detailed analysis of the effects of gait speed on acceleration parameters could provide further insights, especially when considering different speeds within each gait phase (walking and trotting), as previously observed in other studies (5, 25), where speed was measured quantitatively rather than being based solely on gait classification.

The results of the present study provide a solid foundation for future investigations into the biomechanics of canine gait, particularly in the context of different body conformations and health conditions. Next steps would include expanding the sample size to encompass a greater diversity of breeds and body weights, exploring factors such as breed, sex, and age on gait patterns, and comparing healthy dogs with those having clinical conditions, such as hip dysplasia, ligament ruptures, degenerative joint disease, or other orthopedic or neurological pathologies, as previously conducted by researchers studying biomechanics in dogs with dysplasia (22, 27). Changing the environment and surface during analyses, as has also been studied and published in other works (5, 25), could further detail the potential significant impact of these variables on gait.

Finally, we believe that using the results of our study to develop machine learning algorithms could represent a breakthrough in the early detection of movement abnormalities, providing accurate, quick, and cost-effective diagnoses, as anticipated by other authors (17). In the future, real-time monitoring in a natural environment, without human interference, over an extended period, using these algorithms, could provide quantitative data on the impact of these changes on the patients' quality of life, allowing for a clear assessment of recovery or worsening of clinical conditions (21).

## 6 Conclusion

The present study demonstrated that the simplified use of a single triaxial accelerometer positioned in different anatomical regions of the canine body offers a practical and effective approach to gait analysis. Vertical acceleration analysis, combined with analytical techniques such as Fourier Transform, acceleration peak analysis, and autocorrelation, allows for the identification of significant variations in gait patterns between dogs of different body weights.

The results suggest that during trotting, movement shows greater stability, and that the sternum and pelvis regions play a crucial role in stability during locomotion, making them ideal locations for sensor placement. The method used has the potential to be widely applied in biomechanical and clinical studies to monitor canine gait in a more accessible way and in outdoor environments, opening up new possibilities for analysis across different variables, especially when considering sick animals.

## Data Availability

The raw data supporting the conclusions of this article will be made available by the authors, without undue reservation.
